# Depression, Anxiety, and Stress Symptoms (DASS-21) in Elderly Women in Association with Health Status (SHSQ-25): A Cross-Sectional Study

**DOI:** 10.3390/healthcare13010007

**Published:** 2024-12-24

**Authors:** Asta Beniusiene, Vyte Kontautiene, Birute Strukcinskiene, Rasa Grigoliene, Dalia Martisauskiene, Jonas Jurgaitis

**Affiliations:** 1 Faculty of Health Sciences, Klaipeda University, LT-92294 Klaipeda, Lithuania; vyte.kontautiene@ku.lt (V.K.); dalia.martisauskiene@ku.lt (D.M.); jonas.jurgaitis@ku.lt (J.J.); 2 Faculty of Marine Technologies and Natural Sciences, Klaipeda University, LT-92294 Klaipeda, Lithuania; rasa.grigoliene@ku.lt

**Keywords:** elderly women, depression, anxiety, stress, health status

## Abstract

**Background/Objectives:** International studies state that older adults are at an increased risk of mental health symptoms and disorders (depression, anxiety, and stress), especially elderly women aged >65 years. The literature on this topic is scarce, and there is a need for studies that investigate the associations between mental health issues and overall health in elderly women. This study aimed to investigate depression, anxiety, and stress symptoms in elderly women in association with health status. **Methods:** A community-based cross-sectional study was conducted in Klaipeda, Lithuania, in 2020. The survey included elderly women (n = 306) aged 65 to 80 years studying at the Third Age University in Klaipeda city. The DASS-21 and SHSQ-25 scales were used for the survey. Associations and correlations between both scale indicators were calculated. **Results:** Sociodemographic factors such as age, marital status, and place of residence were not statistically significant in terms of mental health, except for women living alone, who had slightly lower levels of depressive symptoms (*p* = 0.015). Mental health issues were quite common; almost half of the participants had higher than normal levels of anxiety (49.0%) and depression (48.4%), and one-third (29.8%) experienced stress. A strong positive correlation was observed between mental health symptoms and physical health, with anxiety having the greatest effect size on fatigue (Cohen’s d = 0.950, *p* < 0.001), cardiovascular symptoms (Cohen’s d = 0.757, *p* < 0.001), and mental status (Cohen’s d = 1.036, *p* < 0.001). Depressive symptoms had a large effect size on fatigue (Cohen’s d = 0.764, *p* < 0.001) and mental status (Cohen’s d = 0.816, *p* < 0.001), while stress had a moderate effect size on all health domains. **Conclusions:** The findings highlight that anxiety symptoms are a major contributor to mental health conditions and overall health in elderly women. This study’s results emphasise the need for targeted interventions to address mental health challenges in elderly women.

## 1. Introduction

Globally, there is a continuous trend in life expectancy, with the proportion of people aged over 80 years growing the fastest [1]. The age and life expectancy of the population are increasing, and the number of health disorders and non-communicable diseases, as well as medical, social, and economic problems, is also increasing. The growth in life expectancy reflects the positive impact of improved social conditions and applied health policies. However, despite living longer, most people are ill or live with a disability for a long time in old age [2]. Cognitive and psychosocial changes are common in older people. As people age, they experience sensory impairment, cognitive decline, frailty, cardiovascular problems, difficulty with daily activities, and an increased risk of falls. These problems are especially common after the age of 85 [3,4]. The health status of older adults is influenced by not only physiological but also social factors, such as loneliness and social isolation [5]. Loneliness has been shown to be particularly important for mental health conditions in the elderly [5,6].

Depression, anxiety, and stress symptoms are the most common mental health problems in older age [7]. Late-life depression is characterised by depressive symptoms in the elderly, associated with mental and physical health challenges. Depressive symptoms are common in older adults with chronic non-communicable diseases. In this case, the functions of older adults are impaired. Depressive symptoms contribute to cognitive decline and are associated with low self-esteem, a loss of interest in daily activities, insomnia, and suicide in the elderly [4,8,9,10]. Depression in an aging population causes somatic symptoms, emotional disturbances, social withdrawal, concentration difficulties, psychomotor retardation, anhedonia, and a loss of energy [11,12,13].

Anxiety in the elderly manifests as fear that occurs when facing a possible threat or unknown situation. This is a normal reaction when faced with danger; however, if it is overwhelming or the feeling persists, then it can manifest as an anxiety disorder. Anxiety disorders negatively affect quality of life [14,15]. Stress in the elderly is characterised by manifestations in the cognitive and emotional scopes [16]. It affects concentration, memory, social relationships, and sleep [12,17,18,19,20].

An estimated 3.8% of the population experiences depression, including 5% of adults (4% of men and 6% of women) and 5.7% of adults older than 60 years. Approximately 280 million people worldwide have depression. Depressive symptoms are present in about one-third of elderly people. According to studies, with increasing age, the course of depression worsens [1,21]. A review analysing studies from 34 countries revealed that the prevalence of depression and anxiety among the elderly could reach up to 19.2%, emphasising the need for enhanced mental health care in this group [22]. Special attention needs to be paid to the increasing prevalence of depression in the elderly, as early detection and early interventions could promote their health and improve their well-being [23]. Depression can cause physical health problems in the elderly, often originating from loneliness. Depressed individuals show poorer health-related behaviours, a risk of cardiovascular disease, and higher levels of inflammatory markers [24].

Previous studies have found that elderly women are at a greater risk of depression than men [4,25,26,27,28]. In a review of 85 studies conducted on all continents, except for Antarctica, strong empirical evidence was found for gender differences in depression among older adults aged 60 or above. Older women scored higher than older men in dimensional measures of depressive symptoms, and older women had higher diagnosis rates of unipolar depression than older men. This pattern was observed in 69 (81%) of the 85 studies [25]. Older women are more vulnerable to negative life events, rumination, and hormonal changes, and they are more affected by psychosocial factors, such as widowhood, living alone, illness, cognitive decline, financial comfort, and caregiving [22,25].

Older adults can suffer from anxiety related to aging. While anxiety is a common experience for everyone, individuals with anxiety disorders often feel intense disproportionate fear and worry accompanied by physical tension and cognitive or behavioural symptoms. These symptoms are difficult to control. If untreated, they can interfere with daily activities, affecting family, social, academic, or work life. It is estimated that approximately 4% of the global population suffers from anxiety disorders. A survey showed that, in 2019, 301 million people worldwide had anxiety disorders, making them the most prevalent mental health issue [29]. Despite effective treatments, only about one in four (27.6%) people suffering from anxiety and requiring treatment receive it. The main barrier to receiving professional support is a lack of information about possible treatments, low awareness, insufficient investment in mental health services, a lack of trained healthcare providers, and social stigma. Some authors call for the need to improve the identification and treatment of anxiety disorders [21,30].

In elderly people, depression and anxiety not only impair mental health but also have a significant impact on physical health. Anxiety and depression, especially their comorbidities, should be managed in elderly people, given their long-term impact on physical disorders and disability. Anxiety and depression are associated with heart disease, hypertension, asthma, vision disorders, persistent cough, gastrointestinal problems, and sleep disturbances [31,32,33].

Factors such as spousal death, widowhood, divorce, loneliness, serious spousal and family member health or behavioural problems, job loss, and financial difficulties can trigger significant stress in elderly individuals. The psychological effect of a death may be a potential factor in the development of chronic stress. In addition, stress may be followed by cognitive decline [18,34,35,36]. Severe and prolonged stress can lead to health problems in elderly individuals. Chronic stress may increase the likelihood of other illnesses and lead to various health complications. Attention should be paid to the harmful effects of stress due to their role in various pathological conditions and diseases. Long-term stress impairs cognitive abilities and is associated with fatigue, emotional disorders, anxiety, and depression [19,20,37,38,39]. Studies examining older adults revealed that stress reduces efforts to be physically active [40].

Chronic non-communicable diseases, long-term treatment, and cognitive decline increase emotional suffering and depression in older people [23]. International studies show that stress and depression need to be consistently investigated because they can cause additional health problems, especially in elderly women who are prone to mental health disorders [22,25,40]. Early identification and targeted intervention to reduce stress and depression could expand prevention possibilities and contribute to the promotion of health and the prevention of chronic diseases [23,41,42,43,44]. It is crucial to investigate mental health disorders (for instance, depression, anxiety, and stress) and overall health, as well as the associations between them.

The aim of this study was to investigate depression, anxiety, and stress symptoms in elderly women in association with health status.

## 2. Materials and Methods

### 2.1. Study Design and Participants

**Figure 1:**
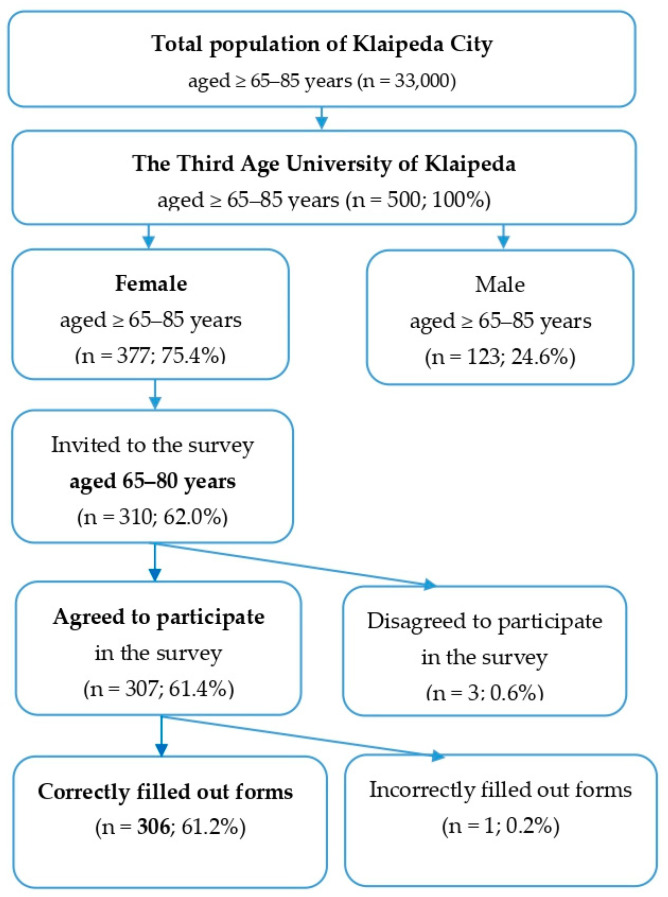
Flowchart of the study participants.

A community-based cross-sectional study was conducted in 2020 in Klaipeda, Lithuania. Elderly people at the Third Age University in Klaipeda (n = 500), aged 65 years or over, were invited to participate in a survey (Figure 1).

Criteria-based sampling was used. Respondents met the following criteria:

Inclusion criteria:

- Female;
- 65–80 years old;
- Attending the Third Age University in Klaipeda;
- Correctly filled out forms.

Exclusion criteria:

- Male;
- Age less than 65 or over 80 years;
- Incorrectly filled out forms.

The male group was excluded from the study due to the small number of participants (n = 123) (it would be incorrect to draw any conclusions from a gender perspective with unequal sample groups of respondents). Elderly women (n = 306) participated in the study. Our respondents were non-clinical participants; they were students at the Third Age University in Klaipeda.

### 2.2. Study Variables

The sociodemographic characteristics of the sample are presented in Table 1. The elderly women (n = 306) were divided into three groups: 65–70 years (n = 155), 71–75 years (n = 93), and 76–80 years or over (n = 56) (Table 1).

The participants were divided into four groups according to marital status: single, married/in partnership, divorced, and widowed. The majority of the respondents were widowed (44.8%, n = 137) or married/in partnership (38.2%, n = 117). The respondents were divided into two groups according to residential status: living alone and not living alone (living with a spouse, partner, children, etc.).

### 2.3. Measurement Tools

Two questionnaires (DASS-21 and SHSQ-25) were used in this study.

DASS-21

DASS-21—the Depression Anxiety Stress Scale—was used to assess the severity of the core symptoms of depression, anxiety (symptoms of psychological arousal), and stress (more cognitive and subjective symptoms of anxiety). The DASS-21 consists of 21 items and is a short version of the 42-item DASS (DASS-42) [45,46,47,48]. This tool was used to measure depression, anxiety, and stress symptoms in elderly women for several reasons: the measure is short, understandable, and easy to use, and it allows for a quick assessment of mental health parameters. The DASS-21 was used to quickly detect potential risks of depression, anxiety, and stress so that elderly women could receive primary psychological support. It did not assess the presence or absence of depression or anxiety. Furthermore, it was not used to clinically diagnose depression or anxiety, but rather to investigate mental and emotional states.

The DASS-21 Lithuanian version has sufficient reliability and, thus, can be used for the Lithuanian population [49,50]. The use of the DASS-21 methodology complies with the requirements of the International and Lithuanian Test Use Regulations [51,52] and professional (medical/psychological) ethics. The individuals who participated in this survey were informed about where and how to receive professional psychological support when necessary.

The development of the DASS-21 was based on differences in the levels of depression, anxiety, and stress symptoms experienced by individuals with normal or more affected levels.

- The depression scale assesses dysphoria, anhedonia, hopelessness, self-deprecation, and a lack of interest and involvement in life, coupled with an overall devaluation of life.
- The anxiety scale assesses autonomic arousal, situational anxiety, and subjective feelings of anxiety.
- The stress scale is sensitive to levels of chronic non-specific arousal. It assesses difficulties in relaxing; nervous arousal; and being easily upset/agitated, irritable/over-reactive, and impatient.

Using a Likert scale, respondents rate each question from 0 to 3 points:

0—Did not apply to me at all (never).

1—Applied to me to some degree or some of the time (sometimes).

2—Applied to me to a considerable degree or a good part of the time (often).

3—Applied to me very much or most of the time (almost always).

Each of the three DASS-21 scales contains 7 items, divided into subscales with similar content. Scores for depression, anxiety, and stress are calculated by summing the scores for the relevant items [53]:

D (depression)—3, 5, 10, 13, 16, 17, and 21.

A (anxiety)—2, 4, 7, 9, 15, 19, and 20.

S (stress)—1, 6, 8, 11, 12, 14, and 18.

To calculate the final score, the DASS-21 scores need to be multiplied by 2. The evaluation score for depression, anxiety, and stress symptoms is divided into 5 levels: normal, mild, moderate, severe, and extremely severe. The severity labels describe the full range of scores in the population. For example, “mild” means that the person is above average, but probably still below the typical severity of someone seeking support [45].

“Normal” is defined as D: 0–9, A: 0–7, S: 0–14.

“Mild” severity is defined as D: 10–13, A: 8–9, S: 15–18.

“Moderate” severity is defined as D: 14–20, A: 10–14, S: 19–25.

“Severe” is defined as D: 21–27, A: 15–19, S: 26–33

“Extremely severe” is defined as D: 28+, A: 20+, S: 34+.

The DASS-21 scores need to be multiplied by 2 to calculate the final score [53].

SHSQ-25

The SHS (suboptimal health status) score was derived from the data collected from the SHSQ-25. The SHSQ-25 includes 25 items on SHS and targets physiological and psychological SHS; it is a reliable and valid instrument for measuring sub-health status [43].

The SHSQ-25 is short and easy to complete; therefore, it is an instrument suitable for use in large-scale studies of the general population. The self-rated questionnaire asks respondents to rate a specific statement on a five-point Likert-type scale based on how often they suffered from specific health complaints in the preceding three months: (1) never or almost never, (2) occasionally, (3) often, (4) very often, and (5) always. The SHSQ-25 highlights the multidimensionality of SHS by encompassing the following domains: (1) fatigue (9 questions: 1–6 and 8–10), (2) the cardiovascular system (3 questions: 11–13), (3) the digestive tract (3 questions: 14–16), (4) the immune system (3 questions: 7, 17, and 25), and (5) mental status (7 questions: 18–24) [54].

Rating of the Likert-scale-assigned points: when summing the scores, the Likert scale scores are converted from 0 to 4 points: 1—0; 2—1; 3—2; 4—3; and 5—4.

The SHSQ-25 score ranges from 0 to 100 points: 0 points indicate the lowest level of health (good health), and 100 points indicate the highest level of health (poor health). The SHS score is calculated by summing the scores for all questions, yielding a total score ranging from 0 to 100 points.

Suboptimal health status is defined as an SHSQ-25 score above 35 points. Scores above 35 indicate SHS, and higher scores represent more severe SHS. The higher the respondent’s SHSQ-25 score, the more severe their suboptimal health status.

According to this, two groups are distinguished: optimal and suboptimal health status. In our study, the respondents were divided into two groups according to their health status: optimal (less than 35 points) health was found in 92.5% (283) of the respondents, and suboptimal (more than 35 points) health was determined in 7.5% (23) of the respondents.

### 2.4. Statistical Analysis

To clarify the appropriateness of the presented research methodology and the reliability of the obtained empirical data, as well as to determine the extent to which different variables of the research instrument measure the same phenomenon, Cronbach’s Alpha coefficient was calculated. For the overall DASS-21, Cronbach’s Alpha was 0.889; regarding the subscales, it was 0.711 for depression, 0.715 for anxiety, and 0.762 for stress (Table 2).

For the SHSQ-25, Cronbach’s Alpha coefficient was calculated to be 0.829. This indicates a high internal consistency. The SHSQ-25 includes five domains. The internal consistency of the SHSQ-25 subscales was not assessed, as Cronbach’s Alpha can only be calculated when a subscale consists of at least 50 items. A statistical analysis was performed using SPSS V.25 for Windows.

A frequency analysis was used to determine the prevalence of mental disorder symptoms in the population under analysis; this was carried out by splitting the symptoms into individual intervals according to the severity of the selected mental problems. Additionally, a descriptive analysis of central tendency measures (mean ± standard deviation (SD)) was used to obtain the gross scores of the data under analysis.

The normality of the variable distribution was tested using the Kolmogorov–Smirnov(a) test [55]. This showed that the data were not normally distributed. Student’s *t*-test (for equal variances) was used to assess the differences between the two categories.

Cohen’s d (d) effect size was also estimated for a better comparison of the effect size in identifying the branches of the research [56]. To estimate the effect size, we applied the norms according to which Cohen’s d and Hedges’ g effect sizes are interpreted: Cohen’s d and Hedges’ g = 0.15—small, 0.40—medium, and 0.75—large effects in gerontology [57]. In cases where the effect size was estimated between different sample sizes, Hedges’ effect was taken into account.

To assess the relationships between the DASS-21 and SHSQ-25 subscales, Pearson’s correlation was calculated. A larger coefficient absolute value described a stronger relationship between the variables in contingency tables. The strength of the correlation was categorised as follows: negligible (0.00–0.10), weak (0.10–0.39), moderate (0.40–0.69), strong (0.70–0.89), and very strong (0.90–1.00) [55,58].

The Kruskal–Wallis statistical criterion was applied when it was necessary to assess the statistical differences between three or more groups (according to age and marital status groups), and the Mann–Whitney criterion was applied when the statistical differences between two groups (according to residential status) were assessed.

All differences were considered statistically significant at a critical level of *p* < 0.05.

## 3. Results

### 3.1. DASS-21 Scores

The mean scores for depression, anxiety, and stress symptoms are the main measures of mental health status in elderly women. The means and standard deviations of the DASS-21 scores are presented in Table 3 according to sociodemographic factors. The data include the depression (D), anxiety (A), and stress (S) scores and the total DASS-21 score. The mean depression score for the examined women was 9.85 ± 6.67, the anxiety score was 9.98 ± 6.97, the stress score was 12.11 ± 7.19, and the total DASS-21 score was 16.00 ± 9.42 (Table 3).

Age

The Kruskal–Wallis test results show no statistically significant differences in the depression, anxiety, or stress scores across the age groups (*p* > 0.05 for all comparisons) The mean depressive symptom scores were 9.76 (65–70 years), 9.84 (71–75 years), and 10.10 (76–80 years), with no significant age-related differences (H(2) = 0.522, *p* = 0.770). The anxiety scores also showed minimal differences across the age groups, although they tended to increase with age: 9.71 (65–70 years), 10.04 (71–75 years), and 10.75 (76–80 years) (H(2) = 1.247, *p* = 0.536). The stress scores were similarly consistent across the age groups: the means for the three age groups were 11.98, 11.69, and 12.92, with no statistically significant difference (H(2) = 0.520, *p* = 0.771). The lack of statistically significant age-related differences suggests that age is not significant in terms of depressive symptoms, anxiety symptoms, or stress levels in the elderly women group.

Marital status

Marital status revealed differences in the mean scores for the DASS-21 indicators, although none were statistically significant. The depressive symptom scores ranged from 8.13 (single) to 10.46 (married/in partnership), but the differences were not significant (H(3) = 6.621, *p* = 0.085). The anxiety symptom scores showed small differences according to marital status, with a mean ranging from 9.46 to 10.39 (H(3) = 3.493, *p* = 0.322).

The stress scores varied slightly across the marital categories (10.66–12.25) and had no significant effect (H(3) = 1.722, *p* = 0.632). It was observed that the married/in partnership participants tended to have slightly higher depression and stress scores than the other groups.

Residential status

The Mann–Whitney U test revealed a statistically significant difference in the depression scores between those living alone and those not living alone (z = −2.429, *p* = 0.015). Women living alone had slightly lower depression scores (9.48 ± 7.30) than those not living alone (10.46 ± 5.47). However, the anxiety and stress scores did not differ much by residential status: the anxiety scores ranged from 9.73 ± 7.63 to 10.39 ± 5.68 (z = −1.849, *p* = 0.064), and the stress scores ranged from 12.02 ± 7.87 to 12.25 ± 5.95 (z = −1.064, *p* = 0.287).

**Figure 2:**
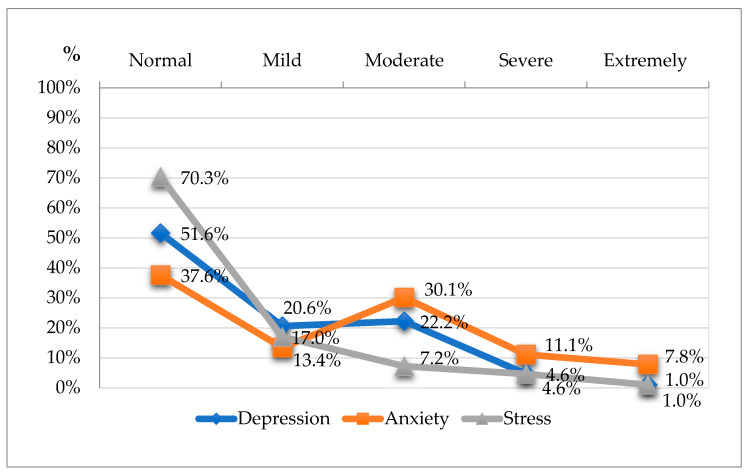
DASS-21 subscale severity ratings.

The results in Figure 2 show the distribution of the research participants across five severity categories—normal, mild, moderate, severe, and extremely severe—offering a detailed view of mental health states. The severity ratings of depression, anxiety, and stress symptoms in elderly women are presented as percentages (Figure 2).

Depression

By analysing the severity of depressive symptoms, it was determined that over half of the participants (51.6%, n = 158) fell within the normal range of depression, indicating no clinical symptoms. Approximately 20.6% (n = 63) exhibited mild depressive symptoms. Quite a large proportion—about one-fifth of the respondents (22.2%, n = 68)—fell within the moderate category, showing more prominent depressive symptoms. Severe depressive symptoms were observed in 4.6% (n = 14), and extremely severe depressive symptoms were observed in 1.0% (n = 3).

Anxiety

The percentage distribution of anxiety severity showed that 37.6% of the participants (n = 115) fell into the normal anxiety range, and mild anxiety symptoms were reported by 13.4% (n = 41). Almost one-third (30.1% (n = 92)) of the respondents were classified within the moderate anxiety range, indicating considerable anxiety prevalence within this sample of elderly women. Severe anxiety symptoms were observed in 11.1% (n = 34), while 7.8% (n = 24) fell within the extremely severe category.

Stress

A stress scores analysis showed that the majority of the participants (70.3% (n = 215)) reported normal levels of stress. Mild stress was reported by 17.0% (n = 52), indicating that a small group experienced low-level stress. Only 7.2% (n = 22) exhibited moderate stress, 4.6% (n = 14) exhibited severe stress, and 1.0% (n = 3) exhibited extremely severe stress.

### 3.2. SHSQ-25 Scores

The SHSQ-25 total and subscale scores are presented in Table 4 according to sociodemographic characteristics, as well as the health status scores of the elderly women. The data include the means and standard deviations of the SHSQ-25 scores, which were analysed according to sociodemographic factors (Table 4).

Sex

The elderly women were found to have a fatigue score of 8.58 ± 4.15, a cardiovascular score of 1.91 ± 1.77, a digestive system score of 1.75 ± 1.49, an immune system score of 2.32 ± 1.62, a mental status score of 7.42 ± 3.69, and a total health SHSQ-25 score of 21.98 ± 9.05.

Age

The Kruskal–Wallis test results showed no statistically significant differences in any of the SHSQ-25 subscales or the total score between the age groups (65–70, 71–75, and 76–80). This suggests that age does not play a significant role in differentiating health status in this sample. Fatigue estimates differed slightly between the age groups: 65–70 years—8.89, 71–75 years—8.07, and 76–80 years—8.66. However, this was not statistically significant (H(2) = 1.448, *p* = 0.485). The scores for the cardiovascular, digestive, immune, and mental systems remained similar and differed statistically insignificantly (*p* > 0.05). The total health scores according to age group ranged from 21.25 ± 9.38 to 22.42 ± 9.26; however, no statistically significant differences were found between the age groups (H(2) = 1.631, *p* = 0.442).

Marital status

Marital status had no significant effect on the SHSQ-25 domains or on the total score: the fatigue scores ranged from 8.05 (divorced) to 8.83 (married/in partnership), with no significant differences (H(2) = 0.625, *p* = 0.732). The scores for the cardiovascular, digestive, immune, and mental systems were similar and did not differ statistically significantly by marital status (*p* > 0.05). The overall health score varied minimally from 20.27 ± 9.90 (single) to 22.63 ± 8.24 (married/in partnership), indicating no significant differences (H(2) = 0.318, *p* = 0.853).

Residential status

There were no statistically significant differences in any of the SHSQ-25 domains or the total health score between those who lived alone and those who did not live alone. On all subscales, except for the immune system, the scores for the health indicator “not living alone” were slightly higher than those for “living alone”, but the difference was not significant (*p* > 0.05). The fatigue scores of 8.43 ± 4.32 (living alone) and 8.82 ± 3.87 (living not alone) (z = −1.137, *p* = 0.256) and the total scores of 21.57 ± 9.52 and 22.63 ± 8.25 were similar and not statistically significant (*p* > 0.05) (Table 4).

In all health status areas, statistically significant differences and large effect sizes were found between the optimal and suboptimal health groups. The key indicators are discussed below. The mean fatigue score for the participants in optimal health was 8.04 ± 3.74, while that for the participants in suboptimal health was significantly higher at 15.21 ± 3.08 (t = −8.940, *p* < 0.001), with a large effect size (Hedges’ g = 1.930). The mean cardiovascular score for the optimal health group was 1.67 ± 1.44 lower than that for the suboptimal group at 4.82 ± 2.67, but the difference was statistically significant (t = −5.592, *p* < 0.001), and the effect size was large (Hedges’ g = 2.01). The total optimal health score (20.52 ± 7.68) was lower than the suboptimal health score (39.83 ± 4.55), indicating a significant difference in overall health (t = −18.334, *p* < 0.001) and the largest effect size (Hedges’ g = 2.575) (Table 5).

Table 6, Table 7 and Table 8 present the SHSQ-25 scores related to depression, anxiety, and stress. This allows for an evaluation of the associations between the SHSQ-25 health domains and the severity of depression, anxiety, and stress symptoms as measured by the DASS-21. These results provide a nuanced understanding of how mental health conditions influence physical health and psychological well-being in elderly women.

The mental status scores were significantly higher in the depression group (8.86 ± 3.44) than in the non-depression group (6.07 ± 3.40), leading to a very large effect size (Cohen’s d = 0.816, *p* < 0.001) (Table 6).

Similarly, the fatigue rates were significantly higher in the depression group (10.11 ± 4.06) than in the non-depression group (7.14 ± 3.71), which was supported by the large effect size (Cohen’s d = 0.764, *p* < 0.001). A similar trend prevailed in terms of the immune system, in that depressive symptoms were associated with a higher number of immune symptoms (2.84 ± 1.73 vs. 1.83 ± 1.33), resulting in a medium effect size (Cohen’s d = 0.655, *p* < 0.001). The effect size for the cardiovascular system was medium but slightly lower (Cohen’s d = 0.449, *p* < 0.001), while the effect size for the digestive system was the smallest (Cohen’s d = 0.263, *p* < 0.001) (Table 6).

The results of the SHSQ-25 domains in the anxiety and non-anxiety groups show that anxiety has a pronounced effect on all health domains. The overall result shows that the anxiety group had a significantly higher total score (25.58 ± 7.95) than the non-anxiety group (15.98 ± 7.48), with a very large effect size (Cohen’s d = 1.244, *p* < 0.001). Mental health symptoms were significantly higher in the anxiety group (8.69 ± 3.46) than in the non-anxiety group (5.31 ± 3.05), and the effect size was large (Cohen’s d = 1.036, *p* < 0.001). Anxiety appeared to correlate strongly with fatigue, with the anxiety group scoring significantly higher (10.11 ± 4.06) than the non-anxiety group (6.50 ± 3.52), as well as having a large effect size (Cohen’s d = 0.950, *p* < 0.001). A similar trend in cardiovascular symptoms was observed: the women in the anxiety group had a higher prevalence (2.37 ± 1.85) of these symptoms than the women in the non-anxiety group (1.15 ± 1.33), resulting in a large effect size (Cohen’s d = 0.757, *p* < 0.001). The other effect sizes were medium: concerning the immune system, the women in the anxiety group had higher (2.67 ± 1.72) scores than the women in the non-anxiety group (1.74 ± 1.22), with a moderate effect size (Cohen’s d = 0.624, *p* < 0.001). The digestive system symptoms were higher in the anxiety group (2.03 ± 1.59) than in the non-anxiety group (1.28 ± 1.19), with a moderate effect size (Cohen’s d = 0.534, *p* < 0.001) (Table 7).

The results of the SHSQ-25 domains in the stress and non-stress groups show that stress has a smaller effect on all health domains such as depression and anxiety. It was found that stress significantly increased fatigue in women, as indicated by the stress group scoring higher (10.25 ± 4.04) in this area than the non-stress group (7.87 ± 4.00), with a medium effect size (Hedges’ g = 0.593, *p* < 0.001) (Table 8).

The stress group was also found to be significantly worse in terms of mental state (9.02 ± 3.29) than the non-stress group (6.74 ± 3.64), which resulted in a medium effect size (Hedges’ g = 0.644, *p* < 0.001). The results show that digestive symptoms were significantly increased in the stress group (2.25 ± 1.59) compared with in the non-stress group (1.53 ± 1.40), with a medium effect size (Hedges’ g = 0.494, *p* < 0.001). The total SHSQ-25 score was higher in the stress group (26.54 ± 8.33) than in the non-stress group (20.05 ± 8.66), resulting in a large effect size (Hedges’ g = 0.758, *p* < 0.001).

The other effect sizes were small: cardiovascular system disorders caused by stress correlated with higher cardiovascular scores (2.40 ± 2.19 vs. 1.70 ± 1.52), evaluated as a small effect size (Hedges’ g = 0.401, *p* < 0.001). The immune system problems associated with stress showed a small effect size on immune system scores (2.62 ± 1.89 vs. 2.19 ± 1.48) (Hedges’ g = 0.267, *p* < 0.001) (Table 8).

### 3.3. Correlations Between DASS-21 and SHSQ-25

A correlation analysis was conducted, and it revealed important insights into the relationships between the DASS-21 (Depression, Anxiety, and Stress Scale) and SHSQ-25 (Health Status Questionnaire) subscales among elderly women (Table 9).

Depression correlations

An analysis of the correlation coefficients indicated that the depressive symptoms in the elderly women were moderately related (r = 0.436, *p* < 0.01) to fatigue and demonstrated a moderate relationship (r = 0.448, *p* < 0.001) with mental status, which suggests a significant link between depressive symptoms and perceived mental well-being (Table 9). Depressive symptoms also moderately correlated (r = 0.304, *p* < 0.01) with immune health, indicating a potential connection between them, though this relationship was weaker than that with the other health indicators. The cardiovascular and digestive system correlations with depressive symptoms were weak (r = 0.291 and r = 0.163), indicating limited associations (Table 9).

Anxiety correlations

The anxiety and fatigue scores revealed a moderate correlation, with a Spearman correlation coefficient (r) of 0.533, which was statistically significant (*p* < 0.01), indicating a relationship between anxiety and physical exhaustion. Anxiety also strongly positively correlated (r = 0.521, moderate) with mental status, emphasising the psychological implications of anxiety (*p* < 0.01). Cardiovascular symptoms were related to anxiety levels with a moderate correlation (r = 0.430, *p* < 0.001). The other systems—the immune system (r = 0.282) and the digestive system (r = 0.222)—exhibited weak correlations, indicating less robust connections to anxiety (Table 9).

Stress correlations

Both mental status and fatigue showed moderate correlations (r = 0.414 and r = 0.409) with stress, implying that stress is associated with mental state and energy levels. The cardiovascular, digestive, and immune systems demonstrated weak correlations (r = 0.201, r = 0.264, and r = 0.185, respectively) with stress, underscoring a weaker but statistically significant relationship between stress and these systems (*p* < 0.01) (Table 9).

## 4. Discussion

### 4.1. Main Findings

In our study, we found that 51.6% of elderly women met the depression norm, 37.6% met the anxiety norm, and 70.3% met the stress norm. This confirms that women who met the norm were characterised by better mental health. The remaining respondents had mental health indicator scores higher than the norm: anxiety was the most common (62.4% in total), followed by depression (48.4% in total) and stress (29.8% in total). Mental health status had a large effect size on fatigue, cardiovascular symptoms, and mental well-being, and a medium effect size on the immune and digestive systems. Anxiety had a large effect size on total health status (the anxiety group had a value of 25.58 ± 7.95, and the non-anxiety group had a value of 15.98 ± 7.48, resulting in a very large effect size (Cohen’s d = 1.244)), as well as on mental status and fatigue. The effect sizes show that depressive symptoms also had a large effect size on mental status and fatigue. Stress had a medium effect size on the above indicators (mental status and fatigue).

Regarding sociodemographic factors, age, marital status, and place of residence did not have statistically significant effects on the prevalence of mental health problems. Women living alone reported slightly lower depressive symptom scores. The participants assigned to the suboptimal health group showed significantly higher SHSQ-25 health status scores in all domains (fatigue, cardiovascular, digestive, immune systems, and mental status).

### 4.2. The Role of Anxiety in Health Status

The results of our study are consistent with those of global and regional studies showing that elderly women are more likely to experience negative emotional health conditions, such as depression, anxiety, and stress symptoms. Previous work suggests that hormonal changes, life events (e.g., widowhood), and societal expectations increase elderly women’s susceptibility to depression and anxiety [25]. This study adds to the scientific literature by linking mental health problems to specific health domains, such as fatigue, cardiovascular, digestive, and immune symptoms and mental status.

Anxiety was the most common mental health disorder, with almost half of the elderly women studied experiencing significant anxiety symptoms. This finding is supported by other studies [30,59,60], indicating that anxiety disorders are common in the older population. Research data show that almost half of the elderly population experiences significant anxiety symptoms [60]. Data from 23 community surveys conducted by World Mental Health (WMH) in 21 countries revealed that 9.8% of 51,547 respondents (response rate = 71.3%) had a 12-month DSM-IV anxiety disorder [30].

We identified a strong association between anxiety and mental state, as well as fatigue, which underscores the interrelationship between psychological distress and physical exhaustion. This is also confirmed by previous research; a specific relationship was identified between psychological anxiety symptoms and SHS (suboptimal health status) [61]. In that study, the authors employed a network analysis to examine the connection between SHS and anxiety symptoms among Chinese residents. “Decreased responsiveness”, “Shortness of breath”, and “Uncontrollable worry” emerged as the central symptoms in the network model. Additionally, “Irritability” and “Exhaustion” functioned as bridge nodes, linking different clusters in the network. This confirms that anxiety can affect various health status domains and the severity of disorders [61].

It is noteworthy that anxiety also showed a moderate association with cardiovascular and immune symptoms, indicating a systemic effect of anxiety on health. Anxiety is an important risk factor for cardiovascular and other common diseases. This is confirmed by other studies demonstrating that anxiety influenced cardiovascular and other common diseases. A previous study found high prevalence rates of depression, anxiety, and stress in cardiac patients [62]. Among cardiovascular patients, the overall prevalence of depression was 31.3% (95% confidence interval: 25.4–38.0%), that of anxiety was 32.9% (95% confidence interval: 21.9–46.6%), and that of stress was 57.7% (95% confidence interval: 45.3–63.3%) [62]. Some studies suggested that anxiety and depressive symptoms were moderately associated with a lower quality of life in people with cardiovascular disorders [63]; therefore, integrating the assessment and treatment of psychological factors into the care of elderly people with cardiovascular system disorders is important. This highlights the benefits of targeted anxiety interventions for the elderly age group [59,63,64,65].

These findings reveal different patterns of mental health severity in elderly women, with anxiety and depressive symptoms being the most common and severe, followed by stress symptoms to a much lesser degree.

### 4.3. The Role of Depression in Health Status

The results confirm that depression has broad relationships across all health domains, including a moderate association with fatigue and immune system symptoms. Depression has a systemic negative influence on overall health. However, there is also a reverse relationship: studies on depression in Chinese older adults show that poor health and multiple chronic diseases are risk factors for depression [66]. In addition, a lack of sleep at night may be one of the causes of mental disorders or consequences of previous mental disorders, and these factors may lead to depression [67].

Depressive disorders are common throughout the life course, and depressive symptoms are present in up to one-third of older adults [1]. In a meta-analysis of 42 articles on depression, conducted by other authors, pooled prevalence estimates based on a random-effects model indicated a depression prevalence rate of 19.2% (95% CI: 13.0–27.5%) [7]. Scientific studies suggest that the prevalence of depression is more relevant in women. For example, the overall prevalence of depressive symptoms was higher among Chinese women [68]. Elderly women tend to score higher on depression measures than older men [25,69].

We found that increased depression scores were significantly associated with higher cardiovascular system scores. This is supported by the results of other authors’ studies. Depressive symptoms were significantly associated with both physical and psychological health status in patients with heart failure. Thus, depressive symptoms are risk factors for poor health status in patients with heart failure [69]. However, increasing resilience may help to improve the psychological health status of patients with depression and heart failure [69]. It is important to note that scientific research shows that depressive symptoms are common in patients with heart failure and have been associated with increased morbidity and mortality [70,71], as well as impaired health status [72].

Some studies have shown that anxiety and depressive symptoms are moderately associated with poorer quality of life. Therefore, integrating the assessment and treatment of psychological factors into the care of older adults with cardiovascular disorders is important [73]. A study conducted in the U.S. not only confirmed the significant burden of disease for major depressive disorder among the U.S. elderly, but also showed an incremental decrease in quality-adjusted life years with the increasing severity of depressive symptoms, as well as significant losses in quality-adjusted life years due to mild depression [73]. The findings of other studies also indicated that anxiety and depressive symptoms were moderately associated with a lower quality of life. Integrating the routine assessment and treatment of psychological factors into the care of older patients with cardiovascular disorders is important for optimising their overall health outcomes and improving their quality of life [63].

Previous studies found that age did not significantly influence the occurrence of depressive symptoms [68,74], and our findings support these results. Studies have suggested that other factors may also influence the occurrence of depression. The prevalence of depressive symptoms was lower among married older adults than among single elderly individuals (i.e., divorced, unmarried, or widowed) [68]. Our study results show that depressive symptom scores did not statistically significantly differ by marital status (single, married/in partnership, divorced, widowed). However, a statistically significant difference was found according to residential status (living alone, living with others) (*p* = 0.015).

### 4.4. The Role of Stress in Health Status

Our study revealed that, although stress had a relatively smaller association with health domains than with anxiety and depression, its moderate correlation with fatigue and mental status highlights its importance in elderly women. The results related to stress are consistent with those of [40], emphasising the cumulative effect of life stressors on an aging population.

Previous studies found that approximately half of the elderly had high-intensity stress symptoms, and, in most participants, behaviours that could negatively affect self-care manifested [75]. Studies by other authors demonstrated a lower degree of stress in elderly people in society. A meta-analysis of stress studies (13 articles reviewed) revealed that stress was experienced by 13.9% (95% CI: 5.5—30.9%) of respondents [7].

The authors studied stress symptoms in elderly subjects and the coping strategies that they used, verifying the relationship between these variables, and they found that elderly individuals may experience stress symptoms due to physical, psychological, and social changes during aging [75]. We found that married/in-partnership participants tended to have slightly higher stress scores than other groups (single, widowed, and divorced).

Stress affects all health domains. Our research found that stress significantly increased fatigue in women, as indicated by the stress group scoring higher than the non-stress group. The stress group was also found to be significantly worse in terms of mental state. Studies by other authors found that stress includes stressful life events, depressive symptoms, and insomnia. Insomnia and stressful life events were the most strongly associated with increased incidence. Some studies showed that stress affects the behaviour and activity of older people [40,76].

### 4.5. Promotion of Mental Health and Well-Being in the Elderly

The WHO’s response to promoting mental health in older people recommends creating a physical and social environment that supports well-being and empowers people to do the things that matter to them. Social activities can significantly improve mental health, life satisfaction, and quality of life, and they can reduce symptoms of depression. Examples of interventions include friendship initiatives, community and support groups, social skills training, creative arts groups, leisure and educational services, and volunteering programmes. The WHO has developed activities to help governments address the mental health needs of older people: *Decade of Healthy Ageing (2021–2030)* (https://www.who.int/initiatives/decade-of-healthy-ageing, accessed on 15 December 2024) and the *Comprehensive mental health action plan 2013–2030* (https://www.who.int/publications/i/item/9789240031029, accessed on 15 December 2024), *Mental Health Gap Action Programme (mhGAP)* (https://www.who.int/teams/mental-health-and-substance-use/treatment-care/mental-health-gap-action-programme, accessed on 15 December 2024), and the *Living with the times* (https://interagencystandingcommittee.org/iasc-reference-group-mental-health-and-psychosocial-support-emergency-settings/living-times-mental-health-and-psychosocial-support-toolkit-older-adults-during-covid-19-pandemic, accessed on 15 December 2024) and psychological interventions have been proposed to address depression, anxiety, and stress [77].

Nowadays, mindfulness-based interventions and mindfulness-based cognitive therapy show a positive effect on depression, anxiety, and stress reduction, and they could also be used in elderly people; in addition, it has been found that animal-assisted interventions (AAIs) reduce the degree of arousal, improve the quality of social interaction, and lift mood. The available results show that AAIs have a positive effect on communication and coping abilities [78,79].

### 4.6. Limitations

The results related to residential status suggest that living alone may be associated with slightly lower depressive symptom scores in elderly women, which may indicate a beneficial aspect of autonomy or self-reliance. Further investigation is required to determine the reasons for this association.

Although, in our study, the associations between depression, anxiety, and stress in elderly women and marital status were insignificant, these aspects could be analysed in future studies, as our findings contradict those of other authors.

### 4.7. Future Research

In this study, we limited the sample to women only. However, future studies could examine the prevalence of depression, anxiety, and stress symptoms in the elderly male population aged over 65 years. In order to understand the mental health of the aging population, it is important to study depression, anxiety, and stress symptoms and other mental health indicators in both genders. Future research could include potential confounding factors, such as chronic disease history.

## 5. Conclusions

This study highlights that elderly women face significant mental health challenges, particularly symptoms of anxiety, depression, and stress, which are closely linked to overall health. Among the elderly women examined, anxiety symptoms emerged as the most prevalent mental health problem, affecting nearly 49.0% of the respondents, followed by depressive symptoms (48.4%) and stress symptoms (29.8%). An analysis of the results revealed that depression, anxiety, and stress symptoms had a significant association with fatigue, cardiovascular disorders, and mental status, with anxiety symptoms showing the strongest associations. Anxiety symptoms not only had a significant association with physical health and fatigue, but also showed a moderate association with cardiovascular and immune disorders. Depressive symptoms also exhibited significant associations, particularly with fatigue and mental status, while the effect size of stress on health domains was moderate. Sociodemographic factors, including age, marital status, and place of residence, were not statistically significant in terms of the mental health domains, although single women reported slightly lower scores of depressive symptoms. Women who were classified as having suboptimal health had significantly higher SHSQ-25 scores in all domains (fatigue, cardiovascular system, digestive system, immune system, and mental status) than those classified as having optimal health. This study’s findings highlight the need for targeted mental health interventions to improve the overall health status of elderly women before their elevated symptom levels of depression, anxiety, and stress reach disease level. This study on depression, anxiety, and stress could provide a useful reference for reducing the risk of depression, preventing stress, and promoting mental health in elderly women. Reducing stress and tension can improve overall health.

## Figures and Tables

**Table 1:** Sociodemographic characteristics.

Variables	Categories	Number	Percentage
Age	65–70	155	51.0
	71–75	93	36.0
	76–80	56	18.4
Marital status	Single	15	4.9
	Married/in partnership	117	38.2
	Divorced	37	12.1
	Widowed	137	44.8
Residence status	Living alone	189	61.8
	Not living alone	117	38.2
Total	Female	306	100

**Table 2:** Cronbach’s Alpha for the DASS-21.

DASS-21			
Depression	Anxiety	Stress	Total
0.711	0.715	0.762	0.889

**Table 3:** Comparison of DASS-21 total and subscale scores according to sociodemographic characteristics.

Variables	Depression Mean (SD)	Anxiety Mean (SD)	Stress Mean (SD)	Total
**Age**				
65–70	9.76 ± 7.22	9.71 ± 7.39	11.98 ± 7.39	15.75 ± 10.03
71–75	9.84 ± 5.86	10.04 ± 6.00	11.69 ± 6.48	15.79 ± 8.01
76–80	10.10 ± 6.53	10.75 ± 7.44	12.92 ± 7.79	16.96 ± 10.10
Kruskal–Wallis	*H*(2) = 0.522	*H*(2) = 1.247	*H*(2) = 0.520	*H*(2) = 0.440
	*p* = 0.770	*p* = 0.536	*p* = 0.771	*p* = 0.803
**Marital status**				
Single	8.13 ± 5.62	9.46 ± 5.52	10.66 ± 6.53	14.13 ± 8.06
Married/in partnership	10.46 ± 5.47	10.39 ± 5.68	12.25 ± 5.95	16.58 ± 7.39
Divorced	9.42 ± 7.16	9.72 ± 7.87	12.17 ± 7.78	15.69 ± 10.44
Widowed	10.27 ± 8.40	9.89 ± 7.83	12.00 ± 8.79	16.08 ± 11.67
Kruskal–Wallis	*H*(3) = 6.621	*H*(3) = 3.493	*H*(3) = 1.722	*H*(3) = 3.888
	*p* = 0.085	*p* = 0.322	*p* = 0.632	*p* = 0.274
**Residential status**				
Living alone	9.48 ± 7.30	9.73 ± 7.67	12.02 ± 7.87	15.64 ± 10.49
Living not alone	10.46 ± 5.47	10.39 ± 5.68	12.25 ± 5.95	16.58 ± 7.39
Mann–Whitney U ^1^	z = −2.429, r = 0.138	z = −1.849	z = −1.064	z = −1.90
	*p* = 0.015	*p* = 0.064	*p* = 0.287	*p* = 0.057
**Total**	9.85 ± 6.67	9.98 ± 6.97	12.11 ± 7.19	16.00 ± 9.42

^1^ In cases where a statistically significant difference (*p* < 0.05) was determined, the effect size r was calculated.

**Table 4:** Comparison of SHSQ-25 total and subscale scores according to sociodemographic characteristics.

Variables	Fatigue Mean (SD)	Cardiovascular System Mean (SD)	Digestive System Mean (SD)	Immune System Mean (SD)	Mental Status Mean (SD)	Total
**Age**						
65–70	8.90 ± 4.25	1.93 ± 1.88	1.81 ± 1.53	2.29 ± 1.64	7.49 ± 3.67	22.42 ± 9.26
71–75	8.07 ± 4.07	1.87 ± 1.68	1.70 ± 1.55	2.25 ± 1.66	7.35 ± 3.88	21.25 ± 9.38
76–80	8.66 ± 4.00	1.95 ± 1.66	1.64 ± 1.31	2.55 ± 1.45	7.28 ± 3.54	22.09 ± 8.05
Kruskal–Wallis	*H*(2) = 1.448	*H*(2) = 0.113	*H*(2) = 0.169	*H*(2) = 3.953	*H*(2) = 0.130	*H*(2) = 1.631
	*p* = 0.485	*p* = 0.945	*p* = 0.919	*p* = 0.139	*p* = 0.937	*p* = 0.442
**Marital status**						
Single	8.27 ± 4.33	2.33 ± 2.35	1.40 ± 1.12	1.80 ± 1.32	6.47 ± 4.14	20.27 ± 9.90
Married/in partnership	8.83 ± 3.87	1.98 ± 1.98	1.80 ± 1.48	2.30 ± 1.47	7.72 ± 3.30	22.63 ± 8.24
Divorced	8.05 ± 4.46	2.22 ± 1.38	1.81 ± 1.76	2.35 ± 1.62	8.10 ± 4.12	22.54 ± 9.86
Widowed	8.55 ± 4.31	1.72 ± 1.58	1.72 ± 1.47	2.39 ± 1.76	7.08 ± 3.80	21.45 ± 9.43
Kruskal–Wallis	*H*(2) = 0.625	*H*(2) = 4.784	*H*(2) = 0.488	*H*(2) = 1.150	*H*(2) = 2.126	*H*(2) = 0.318
	*p* = 0.732	*p* = 0.091	*p* = 0.784	*p* = 0.563	*p* = 0.346	*p* = 0.853
**Residential status**						
Living alone	8.43 ± 4.32	1.86 ± 1.63	1.71 ± 1.50	2.33 ± 1.70	7.23 ± 3.90	21.57 ± 9.52
Living not alone	8.82 ± 3.87	1.98 ± 1.98	1.80 ± 1.48	1.30 ± 1.47	7.72 ± 3.30	22.63 ± 8.25
Mann–Whitney U	z = −1.137	z = −0.014	z = −0.634	z = −0.010	z = −1.256	z = −0.998
	*p* = 0.256	*p* = 0.989	*p* = 0.526	*p* = 0.992	*p* = 0.209	*p* = 0.318
**Total**	8.58 ± 4.15	1.91 ± 1.77	1.75 ± 1.49	2.32 ± 1.62	7.42 ± 3.69	21.98 ± 9.05

**Table 5:** Health status domains in study groups.

Health Status Domain	Optimal (n = 283) Mean (SD)	Suboptimal (n = 23) Mean (SD)	t	*p*	Effect Size Hedges’ g
Fatigue	8.04 ± 3.74	15.21 ± 3.08	−8.940	0.000	1.930
Cardiovascular System	1.67 ± 1.44	4.82 ± 2.67	−5.592	0.000	2.01
Digestive System	1.60 ± 1.35	3.56 ± 1.87	−6.463	0.000	1.405
Immune System	2.13 ± 1.41	4.56 ± 2.23	−5.132	0.000	1.634
Mental Status	7.07 ± 3.53	11.65 ± 2.79	−6.050	0.000	1.315
Total	20.52 ± 7.68	39.83 ± 4.55	−18.334	0.000	2.575

Cohen’s d or Hedges’ g = 0.15, 0.40, and 0.75 used to interpret small, medium, and large effects in gerontology (by Brydges, 2019) [57].

**Table 6:** Comparison of the SHSQ-25 domain scores between the non-depression and depression groups.

Severity	Non-Depression n = 158 (51.6%)	Depression n = 148 (48.4%)	t	*p*	Effect Size Cohen’s d
Fatigue	7.14 ± 3.71	10.11 ± 4.06	−6.682	< 0.000	0.764
Cardiovascular System	1.53 ± 1.50	2.31 ± 1.94	−3.935	< 0.000	0.449
Digestive System	1.56 ± 1.40	1.95 ± 1.56	−2.334	< 0.020	0.263
Immune System	1.83 ± 1.33	2.84 ± 1.73	−5.777	< 0.000	0.655
Mental Status	6.07 ± 3.40	8.86 ± 3.44	−7.129	< 0.000	0.816
Total	18.13 ± 18.26	26.08 ± 8.02	−8.528	< 0.000	0.564

Cohen’s d or Hedges’ g = 0.15, 0.40, and 0.75 used to interpret small, medium, and large effects in gerontology (by Brydges, 2019) [57].

**Table 7:** Comparison of the SHSQ-25 domain scores between the non-anxiety and anxiety groups.

Severity	Non-Anxiety n = 115 (37.6%)	Anxiety n = 191 (62.4%)	t	*p*	Effect Size Cohen’s d
Fatigue	6.50 ± 3.52	10.11 ± 4.06	−7.357	<0.000	0.950
Cardiovascular System	1.15 ± 1.33	2.37 ± 1.85	−6.172	<0.000	0.757
Digestive System	1.28 ± 1.19	2.03 ± 1.59	−4.404	<0.000	0.534
Immune System	1.74 ± 1.22	2.67 ± 1.72	−5.075	<0.000	0.624
Mental Status	5.31 ± 3.05	8.69 ± 3.46	−8.632	<0.000	1.036
Total	15.98 ± 7.48	25.58 ± 7.95	−10.463	<0.000	1.244

Cohen’s d or Hedges’ g = 0.15, 0.40, and 0.75 used to interpret small, medium, and large effects in gerontology (by Brydges, 2019) [57].

**Table 8:** Comparison of the SHSQ-25 domain scores between the non-stress and stress groups.

Severity	Non-Stress n = 215 (70.3%)	Stress n = 91 (29.7%)	t	*p*	Effect Size Hedges’ g
Fatigue	7.87 ± 4.00	10.25 ± 4.04	−4.737	<0.000	0.593
Cardiovascular System	1.70 ± 1.52	2.40 ± 2.19	−3.175	<0.000	0.400
Digestive System	1.53 ± 1.40	2.25 ± 1.59	−3.936	<0.000	0.494
Immune System	2.19 ± 1.48	2.62 ± 1.89	−2.090	<0.000	0.267
Mental Status	6.74 ± 3.64	9.02 ± 3.29	−5.152	<0.000	0.644
Total	20.05 ± 8.66	26.54 ± 8.33	−6.060	<0.000	0.758

Cohen’s d or Hedges’ g = 0.15, 0.40, and 0.75 used to interpret small, medium, and large effects in gerontology (by Brydges, 2019) [57].

**Table 9:** Spearman correlations between the DASS-21 and SHSQ-25 scales and other characteristics.

	Fatigue	Cardiovascular System	Digestive System	Immune System	Mental Status	Depression	Anxiety	Stress
SHSQ-25 Fatigue								
SHSQ-25 Cardiovascular System	0.349 **							
SHSQ-25 Digestive System	0.301 **	0.239 **						
SHSQ-25 Immune System	0.461 **	0.268 **	0.200 **					
SHSQ-25 Mental Status	0.410 **	0.219 **	0.236 **	0.387 **				
DASS-21 Depression	0.436 **	0.291 **	0.163 **	0.304 **	0.448 **			
DASS-21 Anxiety	0.533 **	0.430 **	0.222 **	0.282 **	0.521 **	0.650 **		
DASS-21 Stress	0.409 **	0.201 **	0.264 **	0.185 **	0.414 **	0.623 **	0.639 **	
Residential Status	−0.065	−0.001	−0.036	−0.001	−0.072	−0.139 *	−0.106	−0.038

** Correlation is significant at the 0.01 level. * Correlation is significant at the 0.05 level.

## Data Availability

The data are not publicly available due to confidentiality and privacy considerations. In addition, restrictions apply to the availability of these data due to our policy statement of sharing data. Data are available only upon reasonable request.
